# Intimal and medial contributions to the hydraulic resistance of the arterial wall at different pressures: a combined computational and experimental study

**DOI:** 10.1098/rsif.2016.0234

**Published:** 2016-06

**Authors:** K. Y. Chooi, A. Comerford, S. J. Sherwin, P. D. Weinberg

**Affiliations:** 1 Department of Bioengineering, Imperial College London, London, UK; 2 Department of Aeronautics, Imperial College London, London, UK

**Keywords:** permeability, hydraulic resistance, constitutive modelling, intima, media, artery

## Abstract

The hydraulic resistances of the intima and media determine water flux and the advection of macromolecules into and across the arterial wall. Despite several experimental and computational studies, these transport processes and their dependence on transmural pressure remain incompletely understood. Here, we use a combination of experimental and computational methods to ascertain how the hydraulic permeability of the rat abdominal aorta depends on these two layers and how it is affected by structural rearrangement of the media under pressure. *Ex vivo* experiments determined the conductance of the whole wall, the thickness of the media and the geometry of medial smooth muscle cells (SMCs) and extracellular matrix (ECM). Numerical methods were used to compute water flux through the media. Intimal values were obtained by subtraction. A mechanism was identified that modulates pressure-induced changes in medial transport properties: compaction of the ECM leading to spatial reorganization of SMCs. This is summarized in an empirical constitutive law for permeability and volumetric strain. It led to the physiologically interesting observation that, as a consequence of the changes in medial microstructure, the relative contributions of the intima and media to the hydraulic resistance of the wall depend on the applied pressure; medial resistance dominated at pressures above approximately 93 mmHg in this vessel.

## 1. Introduction

The transport of molecules through the tissues comprising the arterial wall plays an important role in many processes ranging from lipid accumulation in atherosclerosis to contrast agent and drug transport in the diagnosis and treatment of disease. For many of these molecules, the Péclet number (Pe) is substantially greater than 1 [1,2] so their transport is dominated by advection, i.e. they are transported by the bulk flow of water. Studies of the anti-proliferative drug, paclitaxel, have shown the additional complexity that Pe is inhomogeneous through the arterial wall [3]. Understanding such transport requires investigation of the local water transport.

Experimental work on the structural determinants of hydraulic conductance (*L*_p_) has focused on *ex vivo* measurements of water flux across segments of arteries [4,5]. In a few studies, measurements were made before and after selective removal of various wall components, in an attempt to define the causes of the resistance to flow [6]. Interpretation of such experiments is complicated by the collapse of the wall to fill the spaces occupied by the missing components. Measurements have also been made before and after mechanical removal of the endothelium in order to obtain *L*_p_ for the intact and denuded wall and, by subtraction, to estimate *L*_p_ for the endothelium alone. A problem with these methods is that removal of the endothelium may alter properties of underlying layers of the wall by removing a source of vasoactive agents (e.g. endothelin, a vasoconstrictor whose release is pressure dependent) and by altering tissue compaction as a result of the artificially elevated water flux.

To overcome limitations inherent in experimental methods, a number of studies have conducted numerical simulations of water flux across the wall. The geometry of the arterial media is commonly idealized as a regular array of cylinders in two dimensions or three dimensions, approximating the arrangement of smooth muscle cells (SMCs), embedded in porous medium or fibre matrix simulating the extracellular matrix (ECM) [7–9]. These models are mathematically elegant. However, detailed microstructural studies have shown that the media comprises fascicles of irregularly shaped SMCs within a highly structured ECM, in which layers of the fibrous proteins elastin and collagen are embedded in a gel-like ground substance consisting of glycosaminoglycans and proteoglycans [10]. To capture the true anatomy of the media, we recently conducted a combined computational/experimental simulation of water flux in which the medial structure and the permeability of different parts of the ECM were obtained from images of segments of the arterial wall that had been equilibrated with a fluorescent tracer that is restricted to the larger pores through which water flux occurs [11].

When transmural pressure is altered, the medial structure exhibits a nonlinear deformation [12], with cellular reorganization as a result of heterogeneous strain fields arising from aligned solid structures. The dependence of permeability on this deformation is a well-known characteristic of the arterial wall but poorly understood. Experimental observations of diameter and *L*_p_ show nonlinear relationships with pressure [1,5]. Previous studies modelling this dependence [13,14] assumed a relationship between permeability and deformation based on articular cartilage [15] and, as above, used an idealization of the true medial geometry.

In this study, we have extended and modified our combined computational/experimental approach to investigate the effects of pressure on water flux across the arterial media, the whole wall and (by subtraction) the intima. Water flux across the whole wall was measured and image-derived data were used to obtain the shape and distribution of SMCs and the volume and connectivity of the extracellular space at a range of pressures; water flux was modelled in these geometries using values for the ECM permeability coefficient (*k*_ECM_) previously obtained by fibre matrix theory.

## 2. Material and methods

### 2.1. Overview of the combined computational/experimental approach

**Figure 1.**
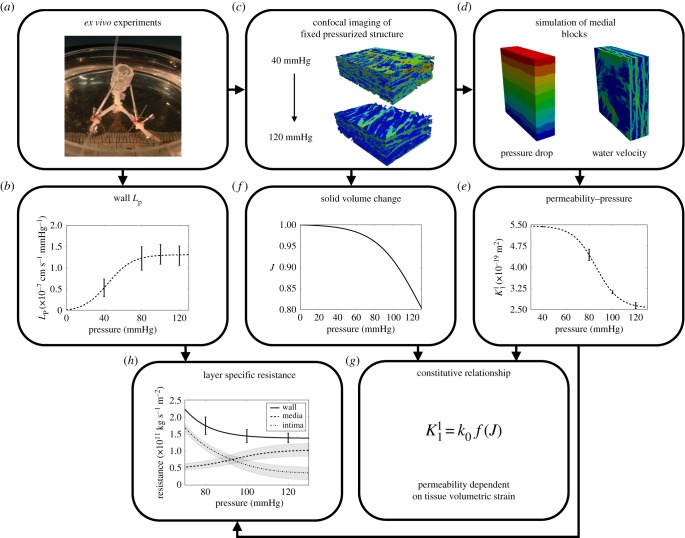
Flowchart of computational/experimental determination of arterial wall transport properties.

Water flux across an *ex vivo* arterial segment of known surface area was measured at a range of pressures and corresponding values of *L*_p_ for the whole wall were calculated (figure 1*a,b*). Albumin labelled with a fluorescent dye that had been added to the working fluid entered the ECM of the wall. Its distribution was mapped by confocal microscopy after *L*_p_ measurements had been completed, following fixation of the tissue at pressure (figure 1*c*). The images were overlaid with a structured computational grid and SMCs were removed from the domain using a penalty parameter. This approach provided realistic geometries of the medial layer for numerical modelling; fluid flow was simulated and the intrinsic permeability of small regions within the media was calculated in all directions, using a permeability coefficient for the ECM obtained previously (figure 1*d*). Performing these simulations for geometries obtained from tissues exposed to a range of transmural pressures related intrinsic medial permeability to transmural pressure (figure 1*e*). Volume fraction change of the medial block within this pressure range was also obtained from the high-resolution images (figure 1*f*). Parametric nonlinear regression fits of these experimental and numerical data were used to obtain a constitutive relationship between medial permeability and solid volume fraction (figure 1*g*). The intimal resistance, *R*_INT_, was determined from wall and medial hydraulic resistances, *R*_WALL_ and *R*_MED_ (figure 1*h*).

### 2.2. *Ex vivo* experiments

#### 2.2.1. Animals

Nine male Sprague Dawley rats (266 ± 6 g; mean ± s.e.m.; Charles River, UK) were housed under a 12 h light cycle at 20–25°C. They were fed a normal laboratory diet (LBS Biotechnology Ltd, UK) ad libitum.

#### 2.2.2. Vessel isolation

The distal abdominal aorta and proximal iliac arteries were cannulated and removed from animals anaesthetized with isoflurane. This segment is of interest because of its propensity for disease [16]. Collapse or over-pressurization of the arteries during the isolation was prevented by a system of reservoirs providing a constant hydrostatic pressure [17,18]. Arterial segment lengths and the bifurcation angle were maintained at their *in vivo* values by tying the cannulae to a stereotactic tripod before removal of the vessels from the body. The whole *ex vivo* preparation was placed into a temperature-controlled bath of Tyrode's Salt Solution (TSS; composition in gram per litre was 8 NaCl, 0.2 KCl, 0.2 CaCl_2_, 0.1 MgCl_2_, 0.05 NaH_2_PO_4_, 1 NaHCO_3_, 1 glucose; pH 6.5) at 37°C that had been pre-equilibrated with 95% air and 5% CO_2_.

**Figure 2.**
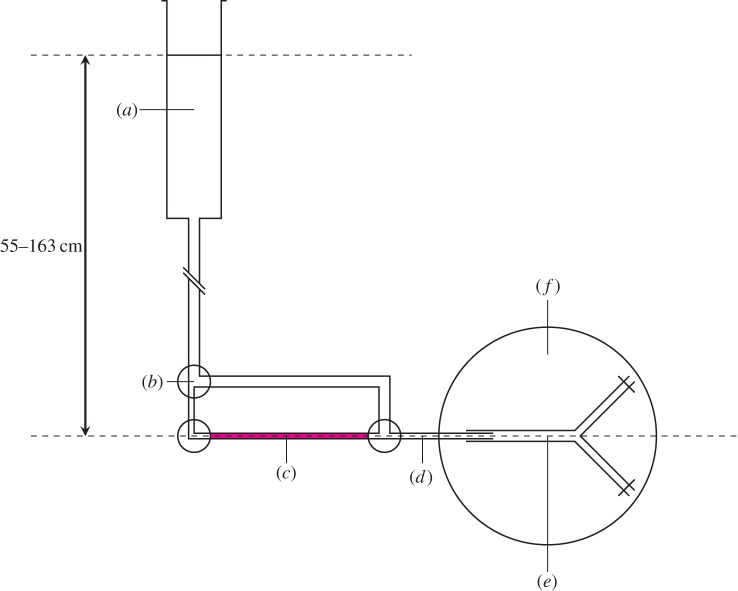
Diagram of *ex vivo* vessel perfusion. (*a*) TSS reservoir above the vessel, (*b*) three-way tap, (*c*) tracer solution, (*d*) graduated capillary tube: inner diameter = 460 µm; length = 30 cm, (*e*) isolated aortic bifurcation: aortic length = 11 ± 0.5 mm, iliac length = 8 ± 0.5 mm and (*f*) temperature-controlled abluminal bath. (Online version in colour.)

Figure 2 shows the system used to perfuse the vessel at pressure *ex vivo*. TSS supplemented with 1% rhodamine-labelled bovine serum albumin (Rh-BSA) and 3% unlabelled BSA was introduced into the lumen and the abluminal TSS was replaced with TSS containing 4% unlabelled BSA.

#### 2.2.3. Whole wall hydraulic conductance

Steady state *L*_p_ was measured at four transmural pressures: 40, 80, 100 and 120 mmHg. Both iliac cannulae were closed to allow measurement of the volume flow rate across the arterial wall by tracking bubble displacement in a graduated tube. The tube was made out of a hydrophobic material in order to prevent bubble slippage [19]. Abluminal fluorescence was monitored over time and steady state transport was observed when fluorescence intensity increased linearly with respect to time; flow rate measurements were then taken for 25 min. Each *ex vivo* preparation was exposed to each pressure once, allowing a steady state to be reached each time, with the order of pressures randomized to account for bias arising from time after isolation. Hydrostatic pressure was changed at a rate of 20 mmHg min^−1^.

The outer surface area, *A*, of the bifurcation was determined from measurements of the arterial segment lengths, diameters and branching angles and *L*_p_ was then calculated
2.1
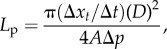
where Δ*x* is the displacement of the air bubble in time step, Δ*t*, within the graduated capillary of inner diameter, *D* and Δ*p* is the transmural pressure across the aortic tissue.

Finally, results were expressed as hydraulic resistance, *R*_WALL_ = 1/*L_p_*.

### 2.3. Microscopy and image processing

#### 2.3.1. Fixation at pressure and embedding

The aortic bifurcations were fixed and dehydrated while maintaining the transmural pressure used for the final *L*_p_ measurement and without removing the vessel from the stereotactic apparatus. The lumen and abluminal surface of the bifurcation were briefly rinsed with TSS and the abluminal bath was replaced with formal sublimate (6% HgCl_2_, 15% formaldehyde) for 30 min. Mercuric chloride acts rapidly (order of seconds) [20] and this fixative also prevented elastic recoil of the vessel when it was released from the apparatus. The vessel was post-fixed in 15% formaldehyde overnight, dehydrated with a graded ethanol series (50%, 70%, 90%, 95% and 100%) and embedded in epoxy resin (EPON 812, TAAB) as described previously [11].

#### 2.3.2. Confocal microscopy

The lateral walls of vessels fixed at each pressure were imaged in three dimensions at a position 2 mm proximal to the apex of the bifurcation (for full details, see Comerford *et al*. [11]). Briefly, embedded arteries were cut in the frontal plane so that the cut face showed a longitudinal section. The cut face was imaged using an inverted laser scanning confocal microscope (Leica, TCS SP5) with the *z*-axis of the z-stack aligned perpendicular to the cut face. Rhodamine fluorescence was excited at 575 nm; emission was imaged at 585–595 nm.

#### 2.3.3. Image processing

**Figure 3.**
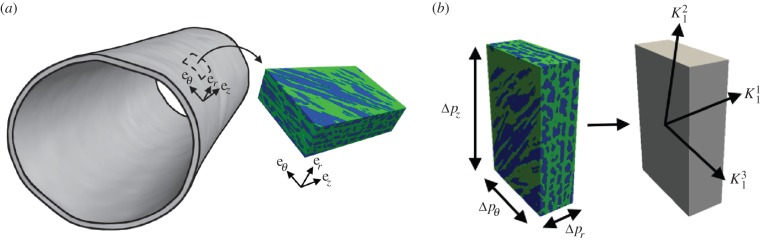
(*a*) Representation of a block of medial tissue and the orientation when it is extracted from the arterial wall. (*b*) Simulation of flow in the realistic medial microstructure (left) due to three coordinate-aligned pressure drops can be reduced to three principal measures of permeability (illustrated on the right): 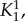 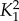 and 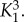

Five cuboidal blocks (figure 3) were extracted from images of three pieces of tissue fixed at each pressure within the physiological range (80, 100 and 120 mmHg.). Additionally, two blocks were extracted from a single tissue specimen fixed at 40 mmHg. Image processing, to correct for intensity attenuation with depth, was performed using Fiji [21] as described previously [11], but with the addition of three image volume rotations to align the imaging axes to the cylindrical coordinates of the aorta.

### 2.4. Effective permeability

Effective permeability refers to the permeability of a hypothetical, uniform region of tissue that exhibits the same overall fluid mechanical properties as a real region of tissue with a non-uniform microstructure. To determine the effective permeability of a porous medium, the flow field must be determined. Flow around solid objects embedded in a porous matrix is described by Brinkman's equation (see Wang & Tarbell [7], Huang & Tarbell [8], Comerford *et al*. [11]). In the arterial media, the solid objects are the SMCs^1^ and the surrounding medium is the porous ECM. In this study, the ECM was assigned an isotropic permeability based on previous models of porous media, which have been validated against experimental data [24,25]; the chosen value, *k*_ECM_ = 1.32 × 10^−18^ m^2^, represents a mean of the reported values. The same value was used at all applied pressures for two reasons: (i) the changes in the volume of the ECM were small, and (ii) the partitioning of water between different compartments of the ECM is complex and it is possible that applied stresses could increase, decrease or not affect the hydration of the part of the ECM through which the majority of water transport occurs. (For example, although increasing the transmural pressure reduced ECM volume, it might have moved water from the relatively impermeable fibrous protein compartment to the more permeable glycosaminoglycan component.) The value of *k*_ECM_ was also increased and decreased by 20% in the analysis to cover variations reported in the literature. Note that the blocks of tissue on which we base our numerical simulations are highly anisotropic—e.g. the SMC is not arranged isotropically and therefore neither is the ECM—and this anisotropy is captured and included in the model. It is the *microscopic* anisotropy of the ECM that is not captured and which we therefore ignore.

We recently outlined a new approach to determine the effective permeability of the arterial media [11] in the open-source spectral/hp element code Nektar++ [26]. Briefly, in this approach we first determine the flow around SMCs in a representative region of the realistic microstructure obtained from confocal imaging data (figures 1*c* and 3*a*). The SMCs are not segmented; rather, the imaging data are directly incorporated into the simulation and regions corresponding to the SMCs are allocated a high resistance. Flow simulations are performed in each of the main directions of the arterial wall (radial, axial and circumferential directions) by applying a pressure drop. From these simulations, we can determine mean volumetric velocity (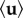) and pressure gradients (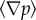). Using Darcy's law
2.2
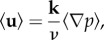
where *v* is the kinematic viscosity and **k** is the permeability tensor,
2.3
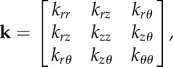
we can form an over-determined system of equations that can be solved using a least-squares approach to find the components of **k**. This tensor can be diagonalized to find the principal components of fluid transport of the arterial wall (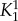 is the approximate radial component—it deviates on average by 3.4° from the radial axis—and 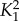 and 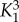 are the two transverse components). Figure 3 illustrates the concept of effective permeability in the arterial wall.

A difference between our previous and present studies is that in the previous one the local concentration of fluorescent tracer was used to estimate the local porosity of the ECM, as well as to identify SMCs, whereas in this study it was only used to identify SMCs. That change was necessary because no transmural pressure gradient was applied in the earlier experiments and hence tracer concentrations could reach an equilibrium; equilibration was prevented by the application of pressure in this study, meaning that the local tracer concentration could reflect transport gradients as well as variation in porosity. The images of fluorescence could still be used to identify SMCs as these completely exclude the tracer. The ECM in which the SMC were embedded was assumed to have a uniform porosity.

The above method to calculate the permeability was used for 17 tissue blocks (to cover the pressure range: 40, 80, 100 and 120 mmHg). For each block, three simulations were performed to extract velocity and pressure data in each of the coordinate directions.

### 2.5. Porous media mixture theory

Hydrated soft biological tissue, such as the arterial wall, can be treated as a mixture of fluid and solid constituents. In the arterial wall, the solid constituents represent the SMC and structural molecules (e.g. proteoglycans, collagen and elastin) and the fluid is water. In figure 3, blue regions represent the impermeable SMCs and the green region is the porous ECM where water flows.

Deformation of soft biological tissue leads to interstitial fluid transport, which can be summarized by the biphasic theory of Mow *et al*. [15]. A small volume (*dV*) of porous medium comprises a volume of solid (*dV*^S^) and volume of fluid (*dV*^F^). This allows us to define the volume fraction (*ϕ*^S^, *ϕ*^F^) of the two components
2.4
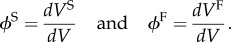
For a saturated mixture, these fractions must obey the saturation condition [27]
2.5
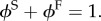
As both the solid and fluid are intrinsically incompressible we can write
2.6
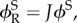
where 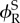 is the volume fraction in the reference state (0 mmHg configuration) and *J* is the volumetric ratio between the deformed and reference configurations. (*J* = det**F**, where the tensor *F* = ∂**x**/∂**X** is the deformation gradient that maps material points (**X**) in the reference configuration to the deformed configuration (**x**)—see Spencer [28].) Volume change is due to fluid entering or leaving a region of tissue.

Several permeability constitutive relationships have been proposed for articular cartilage under deformation (e.g. Holmes & Mow [27]; Ateshian *et al*. [29]; Ateshian & Weiss [30]). Following Ateshian & Weiss [30], we write the constitutive relationship for the permeability (**K**) in the material configuration as
2.7
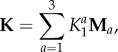
where 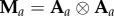 (**A***_a_* representing the unit normal vectors to three planes of symmetry in the tissue) restricts the permeability to three orthogonal planes of symmetry in the arterial wall—radial (*a* = 1) and two transverse planes (*a* = 2, 3)—and 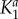 is a function of the relative volume change (*J*), given by
2.8
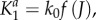
where *k*_0_ is the permeability at 0 mmHg and *f*(*J*) describes how the permeability changes with pressure-driven deformation. (In the arterial wall, the circumferential and axial permeability will also depend on the orientation of the SMCs.) Equation (2.8) is a constitutive relationship that describes the dependence of the intrinsic tissue permeability as a function of deformation, thus characterizing the effect of local volume changes on hydraulic permeability. *k*_0_ and *f*(*J*) can be determined using our method in §2.4 for tissue at different transmural pressures.

### 2.6. Solid volume fraction

#### 2.6.1. Smooth muscle cell volume fraction

The confocal data were transformed onto the quadrature points of the computational mesh (64 000 mesh elements, 8 × 10^6^ quadrature points). Thresholding fluorescence intensities divided the volume into two compartments, the volume occupied by the SMCs (*V*^SMC^) and the remaining volume, corresponding to the ECM. The volume fraction of the SMCs (*ϕ*^SMC^) and ECM (*ϕ*^ECM^) of a medial block with volume *V* can then be defined by
2.9
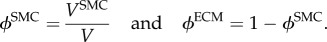
To investigate errors in thresholding and their significance, two observers independently chose thresholds for five images each. The thresholds were found on average to be within 3% of each other and this led to a 2% difference in the measured volume fraction of the SMCs.

#### 2.6.2. Mobile water in the extracellular matrix

To determine the mobile water content of the ECM^2^, experiments were conducted in which porcine aortic tissue was compressed. The tissue samples (*n* = 4; area, 100 mm^2^) were weighed before and during compression between two porous platforms at a pressure of 100 mmHg in a humidified chamber until the compressed tissue mass was constant. Loss in mass was due to loss of water, interpreted as the mobile water. The mobile water content was 37.5% of the initial wet weight.

#### 2.6.3. Solid volume fraction

The solid volume fraction is the solid matrix (e.g. structural ECM fibres, solid components of cells) and water that is not transported e.g. water bound to proteoglycans or inside cells. From our experiments, the mobile water fraction (*ϕ*^W^) for the whole block of tissue is 0.375. The mobile water content of the ECM in the reference state is given by
2.10
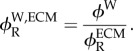
From equation (2.10), we can define a solid volume fraction that varies with pressure
2.11
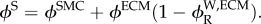
Pore closure is intrinsically included in the value of *ϕ*^SMC^, derived from the confocal data at each pressure, as an expansion of the fraction occupied by SMC would correspond to a reduction in the fraction occupied by ECM and vice versa. Equation (2.11) was evaluated for each pressure (40, 80, 100 and 120 mmHg) and the change in volume fraction of the solid constituents in the wall—*J* in equation (2.6)—was determined.

#### 2.6.4. Radial compression

To provide an estimate of radial compression, the average ECM compression between SMCs and the average SMC thickness within the medial blocks were determined. In this approach, the sum of the distances between SMCs in the radial direction was determined in a one-dimensional measurement across the total block thickness for transmural pressures of 80 and 120 mmHg. The percentage change in ECM thickness was then determined from these two measurements. A similar approach was used to determine the mean SMC radial width: the total thickness in the radial direction of SMCs was determined and divided by the number of SMC layers.

### 2.7. Intimal hydraulic resistance

Using our combined computational/experimental approach, it is possible to determine the mean *L*_p_ of the intima. The arterial wall can be envisaged as an electrical resistor network, where the intimal and medial layers provide resistances in series to water flow that add to give the overall wall resistance, *R*_WALL_ (the adventitia is ignored as it provides little resistance to transport). *R*_WALL_ was obtained from the *ex vivo* experiments described in §2.2.3. This value represents the wall resistance for the entire bifurcation, therefore the following analysis must also be performed over this region. For the medial layer, the permeability can be reformulated as a medial resistance (*R*_MED_) based on thickness (*T*) data from the confocal images and the viscosity of water (*μ*):
2.12
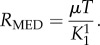
The thickness is an average of the mean medial thickness in the abdominal aorta and iliac arteries (*T* = 36.7 µm). This mean was weighted to account for the different surface areas of the iliac and aortic portions of the bifurcation: 53.8 ± 2% and 46.2 ± 2% of the total area, respectively. The 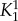 value is the radial permeability component for the blocks extracted from the abdominal aorta. From our data, the iliac arteries exhibit some differences to the abdominal aorta e.g. some breakup of the elastin sheets; however, the overall structure of the SMCs is similar, which is the main influence on radial permeability. For example, at 80 mmHg the structure had a volume fraction of 32 ± 3%, which is similar to the equivalent value for the abdominal aorta of 34 ± 1%. Finally, using these results, *R*_INT_ can be expressed as
2.13
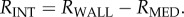


## 3. Results

### 3.1. Hydraulic conductance of the whole wall

**Figure 4.**
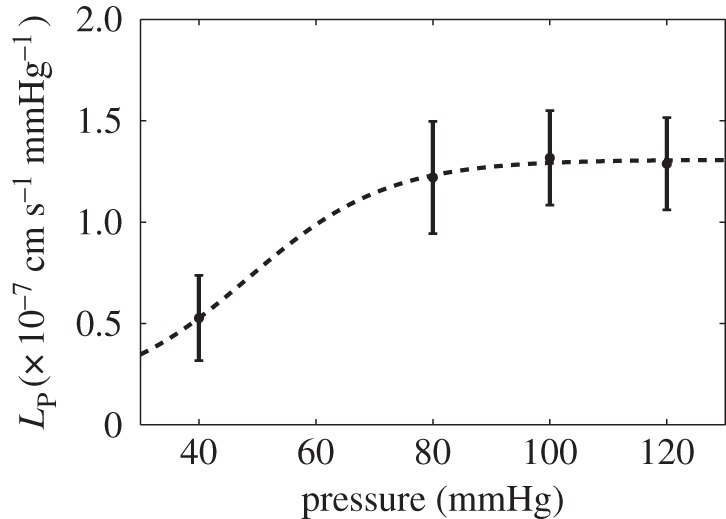
*L*_p_ for the whole wall as a function of transmural pressure difference. Data are represented as mean ± s.e.m. (*n* = 7–8).

Using the methods described in §2.2.3, we measured *L*_p_ as a function of pressure. A sigmoidal function was fitted to the data using parametric nonlinear regression (figure 4). This function was evaluated against a linear fit using the corrected Akaike information criterion (AIC_c_) [31]. The sigmoidal fit had a lower AIC_c_ than the linear.

### 3.2. Permeability of the media

**Figure 5.**
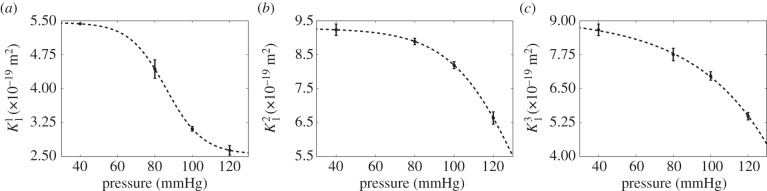
Permeability–pressure relationship in the principal axes of the arterial wall: (*a*) radial component, 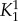; (*b*) transverse component, 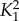; (*c*) transverse component, 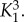

The results for permeability versus pressure obtained from the numerical simulations are shown in figure 5 for the three principal components. The first principal component can be considered the radial component (see ‘principal vectors of the permeability tensor—alignment’ in the electronic supplementary material), while the transverse directions are not the cylindrical or axial coordinates of the artery wall because SMCs are not perfectly aligned with these directions; transport occurs preferentially along the direction of SMC orientation. This is in line with our previous observations that orientation of SMCs alternates through the thickness of the media [11].

The dotted line was determined by parametric nonlinear regression; the fit was only performed in the data range. A sigmoidal function was evaluated against different models (quadratic and cubic polynomials) having a different number of parameters, using the AIC_c_. In all cases, the sigmoidal function had the lowest AIC_c_ and is therefore the preferred model for these data.

### 3.3. Solid volume fraction of the media

**Figure 6.**
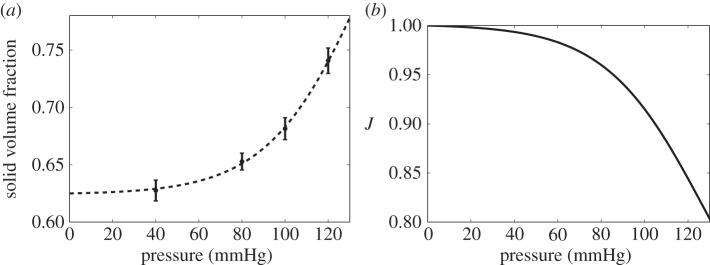
Structural deformation of medial soft tissue due to arterial pressurization: (*a*) medial layer solid volume fraction and (*b*) volumetric change of solid constituents in the media (*J*).

The solid volume fraction of the media is shown in figure 6*a*. As above, it was fitted to a sigmoidal function, enabling the reference solid fraction (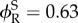) to be determined. The increase in solid volume fraction with pressure is due to compression of the ECM, which drives water out. This can be expressed in terms of the relative volume change (equation (2.6)) from the zero pressure state, shown in figure 6*b*. Between 80 and 120 mmHg, the percentage volume change is 14 ± 1% and the ECM compression in the radial direction (obtained by the methods described in §2.6) was appproximately 5% across the entire medial block.

### 3.4. Constitutive relationship

**Figure 7.**
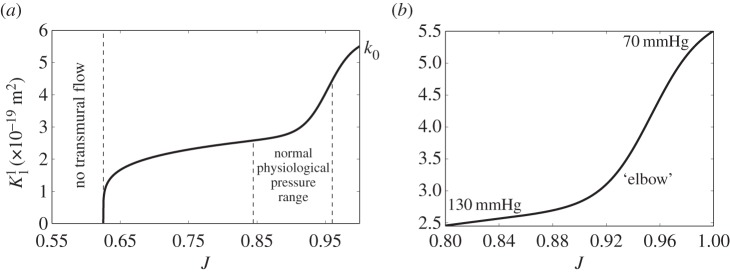
Relationship between radial permeability (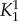) and volumetric change of solid constituents in the media (*J*). (*a*) is the full range and (*b*) is zoomed in on the region corresponding to a pressure range of 70–130 mmHg. The ‘elbow’ represents the region where the gradient of 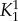 transitions from a steep to a more gradual reduction with pressurization.

For the constitutive relationship, we focus purely on the radial direction, as transverse pressure gradients in the wall are small compared to those in the radial direction. The relationship, shown in figure 7, is described by the following equation
3.1
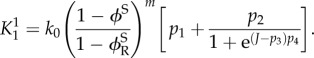

The unknown parameters were determined by parametric nonlinear regression of the permeability and volume change data to be: *p*_1_ = 0.52, *p*_2_ = 0.52, *p*_3_ = 0.95 and *p*_4_ = 55.12. *k*_0_ = 5.51 is a product of the fit. Equation (3.1) can be separated into three distinct parts: the permeability in the reference state (*k*_0_); a conservation constraint, 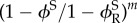 with *m* = 0.2^3^; and the physical behaviour derived from our computational/experimental approach, 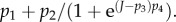 The conservation constraint is a high-pressure physical constraint: when the pores that hold water are fully closed (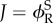), no transmural flow occurs. This would occur only at pressures well beyond the physiological range. The permeability in the physiological range is shown in figure 7*b*. Further details about the constitutive relationship and its finite-element implementation in the open-source software FEBio [32] are given in appendix A.

### 3.5. Mean intimal hydraulic resistance

**Figure 8.**
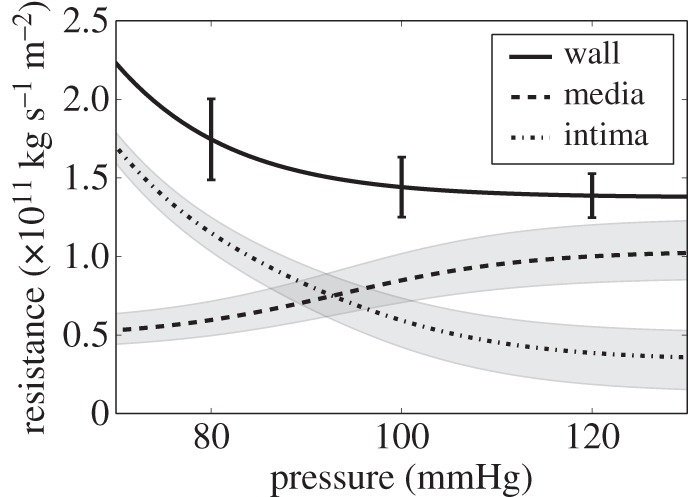
Decomposition of *R*_WALL_ for a two-layered structure. The error bars are s.e.m.s (*n* = 7–8). The grey band around the medial resistance represents a ±20% change in the permeability of the ECM—to cover the range of values reported in literature. The grey band around the intimal resistance represents the propagation of this uncertainty.

*R*_INT_ was calculated using equation (2.13), where *R*_WALL_ was calculated from the experimental total wall *L*_p_ data and *R*_MED_ from equation (2.12). *R*_INT_ < *R*_MED_ for pressures above approximately 93 mmHg (figure 8). In this study, the medial thickness was based on an average thickness for the abdominal aorta and illiac arteries. The measured mean medial thickness was 36.7 µm. An average across all pressures was used as the change in medial thickness with pressure is small—e.g. less than 4 µm in the aorta between 80 mmHg and 120 mmHg. The band on *R*_MED_ and *R*_INT_ represents the effect of allowing the assumed ECM permeability to change by ±20%.

## 4. Discussion

In our *ex vivo* experiments, the hydraulic conductance of the whole wall more than doubled as the transmural pressure difference increased from 40 to 80 mmHg but then remained constant in the 80–120 mmHg physiological range (figure 4). (Note that *L*_p_ is defined as the flow per mmHg pressure gradient and that it accounts for changes in surface area but not wall thickness; if it remained constant as pressure increased from 80 to 120 mmHg that would mean that the flow per unit surface area of the wall increased 50%.) The dependence of aortic *L*_p_ on pressure has varied between previous studies: like us, Baldwin *et al*. [19], Baldwin & Wilson [33] and Shou *et al*. [5] obtained a plateau over the physiological range, but Tedgui & Lever [17] found a substantial decrease in *L*_p_ as pressure increased; Baldwin & Wilson [33] and Shou *et al*. [5] showed a decrease in *L*_p_ from sub-physiological to physiological pressure, whereas Baldwin *et al*. [19] found *L*_p_ was constant over this range. (And at 100 mmHg—the centre of the physiological range—the study of Shou *et al*. [5] gave an *L*_p_ approximately fivefold lower than the one obtained here, presumably reflecting their use of the rat thoracic aorta rather than the thinner terminal aorta and proximal iliac arteries.)

This variation may reflect the complexity of the physical interaction between pressure and wall thickness. Increased pressure will distend the vessel and thus will tend to reduce wall thickness. The extent of the distension will depend on the material properties of the wall; the thoracic aorta used in the four studies listed above is likely to be more elastic than the abdominal segment used here. The reduction in the wall thickness will also be affected by any changes in hydration that result from the distension. Wall thickness will also be affected by solvent drag, but the direction is hard to predict. Considering flow through a compressible sponge, analogous to the wall tissue, the sponge will be stretched by solvent drag if it is anchored at its upstream surface but will be compacted if it is anchored at its downstream surface. Because of the concentric layering of more and less distensible components in the wall, the actual response of the aorta to increased transmural flow is not intuitively obvious. Net effects of all these factors, and of endothelium-derived vasoactive agents, on wall thickness have been measured: Baldwin *et al*. [19] found a decrease in wall thickness as pressure was increased from 50 to 100 mmHg, but no further change when pressure was increased to 150 mmHg.

Medial permeability was computed using microstructures obtained from confocal images of tissue fixed at different pressures: mapping the uptake of a fluorescent extracellular fluid-phase marker in three dimensions allowed separation of the tissue into SMC and ECM compartments with sub-micrometre spatial resolution, avoiding the assumptions of idealized medial architectures in earlier work. The simulations required no free parameters and only one value from the literature: the average permeability of the ECM. Several values have been reported [7,8,34], so it is possible that the actual ECM permeability differs slightly from the chosen value. The shape of the 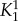 versus pressure curve would not alter if the value were changed, but the absolute magnitude would. The former was confirmed by increasing and decreasing the value of *k*_ECM_ by 20%. The calculated medial resistance consistently lay below the experimentally measured total wall resistance at all pressures, so the magnitudes are at least plausible.

The simulations showed that *K* in all three principal axes decreases substantially as pressure is increased within the physiological range. Variability between simulations based on different samples obtained at the same pressure was small, and the changes with pressure were large, supporting the view that the trends with pressure are reliable. Deformation of the arterial wall under increased pressure causes a reduction in 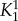 through ECM compaction. This compaction, resulting from pore closure, was evident visually in the confocal data and confirmed by computing *ϕ*^S^ and *J*: as pressure increased from 80 to 120 mmHg, the volume reduction of the ECM was 14 ± 1%. Radial compression of the ECM, obtained by the method described in §2.6, was approximately 5% over the same pressure range. The SMCs, which can be considered nearly incompressible, are brought into closer proximity to one another due to ECM compaction. Additionally, the cells become thinner due to stretching. Between 80 and 120 mmHg, the mean number of SMCs per block in the radial direction increased from 3.47 to 4.34 and the mean cell width decreased from 2.11 to 1.83 µm. This further increases the resistance to paracellular water transport.

These observations are captured in our proposed constitutive relationship, which for the first time relates radial permeability to strain driven by ECM compaction (figure 7). The relationship between the permeability of soft biological tissues and volume change (or volumetric strain) is well known in the realm of articular cartilage and ligament mechanics [27,29,30,35]. In this study, narrowing of intercellular channels accounts for more than approximately 80% of the permeability change within the physiological range, justifying our assumption that the ECM permeability is constant with pressure. Note that the ‘elbow’ in figure 7*b* is not an artefact arising from the fitting of a sigmoidal function to the permeability data (figure 5*a*)—it arises from a similar kink in the relationship between pressure and the change in solid volume (figure 6*a*). Our results differ from previous models and assumptions [13,14,36] presumably because we derived our deformation relationship from experimental data and took into account image-derived aspects of the medial microstructure.

Our image-based numerical simulations also demonstrated that medial permeability varies with direction and that this anisotropy is pressure dependent. At 80 mmHg, the permeability in the transverse direction was two times larger than that in the radial direction; at 100 mmHg, this ratio increased to 2.6 but at 120 mmHg it reduced to 2.5, suggesting that additional pressurization might further reduce anisotropy. *In vivo*, the largest pressure gradient occurs from the luminal to the abluminal side. Transverse components are significantly smaller, but they can rise in areas where geometrical undulations and uneven wall deformation occur.

The directionally varying trend can be explained as follows: ECM compaction causes 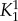 to reduce at a lower pressure than 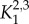 due to intercellular channels narrowing preferentially in the radial direction. In the transverse directions, the tissue stiffens preferentially along principal directions related to the orientation of collagen [37]; collagen and SMCs have similar orientations in the arterial media [38]. Combined, these effects mean that the relative ease of water transport in the transverse directions is elevated due to SMC alignment; however, radial transport will always dominate in the arterial wall due to the transmural pressure gradient.

A number of experiments have attempted to determine endothelial *L*_p_ by comparing measurements of water flux obtained before and after removal of the endothelial layer [4,17]. As already noted, the unstated assumption that *L*_p_ for the rest of the wall is unchanged by this procedure may be incorrect because a source of vasoconstrictors and vasodilators has been removed and because solvent drag is increased. An alternative approach has been to investigate *L*_p_ of endothelial monolayers cultured on porous substrates *in vitro* [39,40]. The drawback of this method is that the artificial environment may degrade the barrier properties of the endothelium. It is known that these monolayers have elevated permeability to small proteins such as albumin, which, like water, are transported through intercellular junctions.

In this study, the combination of experimental and numerical techniques gave *L*_p_ for the whole wall and for the media. Hence, *L*_p_ for the intima could be obtained by subtraction; intimal, medial and total wall resistances are obtained from fully intact tissue with this method. The implicit assumption that all the adventitia was removed when the vessel was cleaned is unlikely to be critical because the adventitia has a loose structure that is not expected to offer much resistance to water flux. The results presented in figure 8 demonstrate that the intima provides the largest contribution to *R*_WALL_ below approximately 93 mmHg while the medial layer dominates above approximately 93 mmHg^4^. At 80 mmHg, the medial layer represents approximately 40% of the *R*_WALL_, while at 120 mmHg it represents approximately 80%. (Shou *et al*. [5] obtained approximately 50% for all pressures from 80 to 120 mmHg.) This dominance will probably increase for thicker walled arteries, in which the media contributes a higher percentage of the wall thickness. Intimal resistance appeared to decrease with pressure. We hypothesize that this results from pressure-induced stretch of the endothelium and the resulting shortening of the intercellular junctions. Stretch of the endothelium is known to increase permeability to albumin [41].

The methods applied in this study could be used to quantify other transport properties of tissue, such as diffusivity. Recent work by Levin *et al*. [42] showed for various drugs and macromolecules that transmural and transverse diffusivities in segments of the calf carotid artery were significantly different. Using our computational/experimental approach, we could characterize anisotropy of drug/macromolecule diffusion in more specific locations (e.g. at sites prone to, or protected from, disease) and understand how microstructural reorganization affects diffusivity.

The methods have a number of limitations. First, arteries *in vivo* are exposed to pulsatile pressures but both our experimental and numerical studies used steady pressures. It is unclear how cyclical pressure changes would affect the microstructure of the wall. Second, we have treated the ECM as a homogeneous medium whereas it is actually composed of different compartments of differing resistance to water flow and—potentially—different levels of water loss under applied pressure. Third, for practical reasons, the computation of the medial permeability was limited to blocks taken from the left and right side of the abdominal aorta. Ideally, blocks would be sampled over many different tissue regions e.g. in the iliac arteries and the inner and outer walls of the abdominal bifurcation. Unfortunately, from a computational perspective this is still challenging, given the need for repeated sampling and three simulations to determine the permeability tensor (in this study the total number of core hours totalled 73 440 for the 17 tissue blocks). Furthermore, to have sufficient accuracy in the experimental measurements, the hydraulic conductance had to be measured over a sufficiently large area of artery. As advances are made in experimental methods, smaller more localized tissue regions will be considered.

## Supplementary Material

ESM

## Figures and Tables

**Figure 9.**
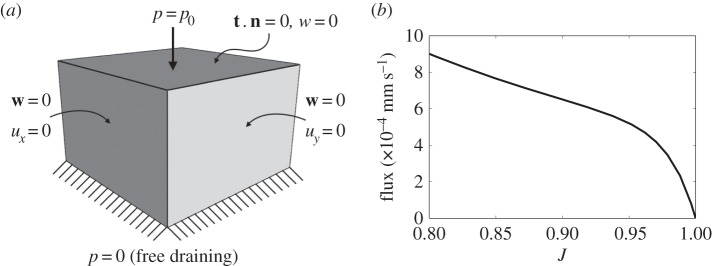
(*a*) Boundary conditions for the biphasic analysis. **w** is the water flux, *p* is the pressure, **t** is the traction vector, **n** is the normal vector and *u* the displacement. (*b*) Water flux variation with volume as the medial tissue is compressed. Data extracted from an element approximately 10 µm from the fixed surface.

## Endnotes

1 Shanahan *et al.* [22] showed that a SMC immersed in a solution that is hypertonic by 100 mOsm (equivalent to a pressure of −1930 mmHg) will lose 8% of its volume in 5 s. Using a typical SMC length, diameter and volume from Todd *et al.* [23], and for simplicity assuming a cylindrical geometry and that the cell membrane has a much higher resistance to water flux than the cytoplasm, we compute *L*_p_ = 2 × 10^−10^ cm s^−1^ mmHg^−1^ for a cell. If the whole wall (approx. 5 laminae thick) was composed of cells, it would have *L*_p_ = 10^−11^ cm s^−1^ mmHg^−1^. That is many orders of magnitude lower that the *L*_p_ observed for the whole wall in the present study and hence it is a reasonable simplification to assume that the cells are impermeable.

2 Extrafibrillar extracellular water.

3 The choice of *m* is arbitrary—it describes how the curve transitions to zero when all water has been exuded.

4 The crossover point has an uncertainty based on the ±20% ECM permeability. In this study, the range was from 86 to 101 mmHg.

## Appendix A. Finite-element implementation of the medial layer permeability constitutive relationship

This section shows how the developed constitutive relationship can be implemented in the open-source finite-element software, FEBio [32]. Medial permeability as a function of volumetric deformation is given by equation A 1.

A 1

with the parameters defined in table 1 for the different directions in the wall. Following the method of Ateshian & Weiss [30], to evaluate the permeability in a nonlinear finite-element formulation, the permeability must be linearized and thus the rate of change of permeability with deformation must be evaluated. If we assume that the tissue in the material frame is governed by

A 2

then the rate of change of permeability in the material frame is given by

A 3

 can be expressed in the spatial frame as

A 4

where *I*_3_ = *J*^2^ and **I** is the identity matrix. Substituting equation (A 1) into equation (A 4) gives

A 5

To demonstrate how medial compression relates to water flux, a cuboidal region (50 × 80 × 80 µm in the radial, axial and circumferential directions, respectively) was created and discretized using PreView (www.febio.org). A biphasic analysis was then undertaken in FEBio [32]. For this analysis, the medial tissue consisted of a solid matrix (neo-Hookean material, *E* = 0.1 kPa and *v* = 0.4) and a permeating fluid with a hydraulic permeability governed by equation (A 5). The tissue was compressed in the radial direction by fixing one end and applying a fluid pressure to the other. The fixed bottom surface was allowed to drain freely, while all other surfaces were impermeable. The lateral walls were fixed, except for the radial displacement, to restrict compression to the radial direction. The applied pressure gave a maximum radial displacement of 4.6% in the physiological range. A summary of boundary conditions is given in figure 9*a*. Figure 9*b* shows the flux of water in the block at a location approximately 10 µm from the fixed surface as the tissue is compressed. The shape of the flux curve is indicative of the permeability constitutive relationship: initially the permeability is higher which with application of pressure causes the flux to increase. As the fluid is exuded the permeability reduces but does not drop rapidly with volume. This is seen by a reduced gradient of fluid flux.

**Table 1.** Material coefficients for different directions in the arterial wall. Asterisks, not material parameters.

direction	*p*_1_	*p*_2_	*p*_3_	*p*_4_	*k*_0_*	*m**
	0.52	0.52	0.95	55.12	5.51	0.2
	0.36	0.68	0.82	15.29	9.29	0.2
	0.63	0.39	0.90	27.20	8.43	0.2
