# Supplementary material for: Intimal and medial contributions to the hydraulic resistance of the arterial wall at different pressures: a combined computational and experimental study
Source: J R Soc Interface. 2016 Jun;13(119):20160234. doi: 10.1098/rsif.2016.0234 (PMC4938088; doi:10.1098/rsif.2016.0234)
Supplement: ESM [file rsif20160234supp1.pdf]

Supplementary Information

Principal vectors of the permeability tensor - alignment

For the radial direction, which is well defined (being perpendicular to the endothelium), the results are consistent - directions do not change with pressure (Table 2). The other two principal directions, which are less well defined, are consistent within a floating frame of reference - i.e. within each block the numbers are highly consistent.

Table 2: Absolute angle between principal permeability directions and the cylindrical coordinates of the arterial wall

	Block	Radial direction	Axial direction*	Circumferential direction*
80 mmHg	1	2.6°	19.1°	19.1°
	2	4.4°	19.8°	20.3°
	3	2.6°	17.9°	18°
	4	4.4°	10.7°	10.7°
	5	2.6°	2.0°	2.8°
100 mmHg	6	2.6°	31.2°	31.1°
	7	2.6°	50.2°	50.2°
	8	2.6°	8.5°	8.6°
	9	2.6°	15.9°	16.6°
	10	4.8°	1.4°	2.0°
120 mmHg	11	4.4°	29.8°	29.4°
	12	3.6°	59.0°	58.9°
	13	2.6°	5.2°	5.3°
	14	3.6°	7.0°	7.0°
	15	5.1°	22.6°	22.1°

*Alignment in the transverse plane of the wall has no fixed reference as the confocal images may be rotated; hence it cannot be easily interpreted

Fixation of tissue in deformed state

In order to obtain realistic images of wall structure in vessels subjected to physiologically relevant transmural pressures, it was necessary to obtain fixation of all the elastic components of the wall while still in the stressed configuration. Otherwise, elastic recoil once the pressure is removed would have introduced artefacts into the images e.g: wavy or wrinkled elastic lamellae in the tunica media. Elastin is difficult to fix with common aldehyde fixation (Fung and Sobin, 1981), retaining its elasticity even after prolonged fixation in a deformed state. A comparison of common fixatives was conducted to select a suitable one. Formal sublimate (FS) was prepared by making a saturated solution of $HgCl_2$ in 15% formaldehyde. As the elasticity of elastin is attributed to hydrophobic interactions of its component amino acids, dehydration in the strained state was also trialled with each fixative.

The original length (l_o), length during fixation (l_d) and final length after fixation and dehydration (l_a) of each sample were recorded and the results expressed as percentage of strain retained after fixation and dehydration ie: Percentage of strain fixed= $\frac{\frac{l_a - l_o}{l_o}}{\frac{l_d - l_o}{l_o}}$. Results are shown in Figure 10.

Sample bubble displacement data

Bubble speeds were recorded in the range (0.46mm min^{-1}, 3.25mm min^{-1}) at 40mmHg and 120mmHg respectively (Figure 11).

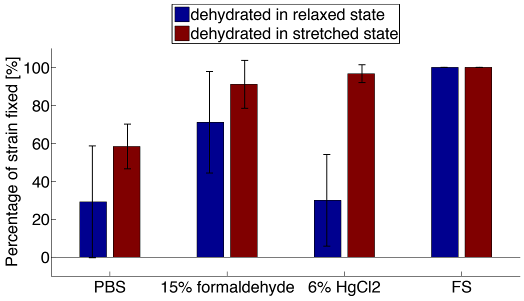

Figure 10: Results of fixation and dehydration trials: dehydration of stretched samples resulted in less recoil for all fixatives. FS performed best of all the fixatives, preserving 100% of the strain.

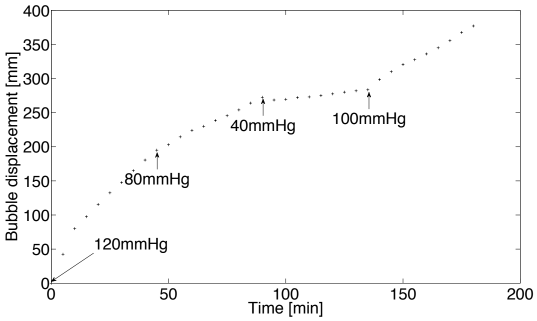

Figure 11: Typical displacement trace of bubble in flux measurement graduated tube. In this case, the bifurcation was placed under transmural pressures in the following order: 120mmHg, 80mmHg, 40mmHg and 100mmHg. Changes in pressure are indicated by arrows. The 'sealing' phenomenon previously reported (Baldwin et al., 1992) is observed in this trace within the first 15 min.
